# Improvement of Cardiovascular Risk Factors by Genistein Supplementation: A Systematic Review and Meta-Analysis in Diverse Population-Based RCTs

**DOI:** 10.1155/jnme/1827252

**Published:** 2025-03-18

**Authors:** Hanxiao Feng, Kuan Jiang, Yi-feng Zhang, Jinhong Zhuang, Cun Ku, Jinzhao Yang, Yang Zhang

**Affiliations:** ^1^School of Public Health (Shenzhen), Sun Yat-Sen University, Shenzhen, Guangdong, China; ^2^Guangdong Provincial Key Laboratory of Diabetology, Guangzhou Key Laboratory of Mechanistic and Translational Obesity Research, The Third Affiliated Hospital of Sun Yat-Sen University, Guangzhou, Guangdong, China; ^3^GuangDong Engineering Technology Research Center of Nutrition Transformation, Sun Yat-Sen University, Shenzhen, Guangdong, China

**Keywords:** cardiovascular disease, genistein, meta-analysis, randomized controlled trials

## Abstract

Genistein[5,7-dihydroxy-3-(4-hydroxyphenyl)chromen-4-one] is a phytoestrogens known to positively impact various cardiovascular disease (CVD) risk factors. However, not all studies have yielded consistent results, and existing meta-analyses have not comprehensively addressed all CVD risk factors. We conducted a systematic search of the PubMed, ISI Web of Science, Embase, and Cochrane Library databases up to June 2024, following PRISMA 2020 guidelines. We included adult randomized controlled trials (RCTs) that examined pure genistein supplementation without other combined interventions and reported on at least one CVD risk factor. Data extraction and quality assessment were performed independently by two authors using a standardized form and the Cochrane Collaboration Scale. A total of 21 RCTs were included, with 941 participants in the genistein supplementation group and 918 participants in the control group. Statistical analyses were conducted using R software with the meta package. The meta-analysis revealed that, compared to the placebo group, genistein supplementation significantly improved the levels of TC ([MD 95% CI: −9.38 [−14.64, −4.12]; *p* < 0.001]), LDL-C ([MD 95% CI: −11.14 [−19.42, −2.86]; *p* < 0.001]), Lp(a) levels ([MD 95% CI: −0.69 [−0.98, −0.41]; *p* < 0.01), SBP ([MD 95% CI: −8.32 [−12.44, −4.20]; *p* < 0.01), DBP ([MD 95% CI: −3.57 [−5.25, −1.89]; *P*=0.04]), fasting blood glucose ([MD 95% CI: −3.98 [−6.79, −1.17]; *p* < 0.001]), fasting insulin ([MD 95% CI: −1.79 [−2.05, −1.54]; *p* < 0.01), HOMA-IR ([MD 95% CI: −0.56 [−0.64, −0.49]; *p* < 0.01), and homocysteine levels ([MD 95% CI: −0.74 [−1.05, −0.42]; *p* < 0.01). However, there were no significant improvements in TG, HDL-C, and CRP levels. The observed improvements align with clinically meaningful thresholds for cardiovascular risk reduction. Substantial heterogeneity observed for most outcomes was explored via subgroup analysis. Subgroup analyses were conducted based on treatment duration, geographic region, or participant health status, and heterogeneity was assessed using the *I*^2^ statistic. Subgroup analysis did not reveal any significant differences, indicating that heterogeneity was not influenced by factors such as treatment duration, geographic region, or participant health status. Overall, this meta-analysis provides consistent evidence that genistein intake significantly reduces several important CVD risk factors, including TC, LDL-C, Lp(a), SBP, DBP, fasting blood glucose, fasting insulin, HOMA-IR, and homocysteine levels.

## 1. Introduction

Cardiovascular disease (CVD) is widely recognized as the leading cause of mortality, posing a significant threat to human health and accounting for a substantial proportion of global noncommunicable disease-related fatalities. CVD is associated with various risk factors, including an unhealthy diet, tobacco smoking, lack of physical activity, dyslipidemia, hypertension, diabetes, obesity, and inherited genetic susceptibility [1].

While conventional medications (e.g., statins and antihypertensives) are widely used and effective, there is growing interest in natural compounds and herbal medicines that may offer complementary benefits, including fewer side effects and the potential to target multiple metabolic pathways. Polyphenols, flavonoids, and phytochemicals have been identified as crucial bioactive compounds in the prevention of CVD. Genistein is a bioactive isoflavone abundantly found in legumes like soybeans, fava beans, and chickpeas. As a phytoestrogen, it can bind to estrogen receptors and exert estrogenic effects [2]. It also activates peroxisome proliferator-activated receptors (PPARs) [3, 4], exhibits redox activity [5, 6], and inhibits tyrosine kinase [7, 8]. Notably, genistein has shown the potential in alleviating mitochondrial dysfunction which is associated with metabolic disorders and certain aspects of CVD pathophysiology [5, 6]. Mitochondrial dysfunction, characterized by abnormalities in the respiratory chain and impaired ATP synthesis, can contribute to endothelial injury, oxidative stress, and insulin resistance—factors closely linked to CVD [5]. While genistein has shown promise in several pathophysiological processes, there remain challenges in fully understanding its mechanisms, particularly in how it modulates cardiovascular health through lipid metabolism and blood pressure regulation.

In the cardiovascular system, genistein demonstrates potential health advantages. Studies have shown that daily supplementation with genistein can enhance cardiac function and reduce metabolic and cardiovascular risk factors in postmenopausal women with metabolic syndrome [9, 10]. In vitro studies suggest that genistein can inhibit cellular cholesterol efflux and restore impaired endothelial function in the aorta [11, 12]. Numerous animal studies have explored the pathophysiological benefits and mechanisms of genistein. For instance, the administration of genistein has been shown to increase glucokinase and insulin levels in rats with Type 1 diabetes [13], alleviate vasoconstriction by restoring nitric oxide–mediated signaling [14], improve cardiac dysfunction caused by excessive activation of the renin–angiotensin system [15], and mitigate cardiac fibrosis in rats with Type 1 diabetes [16].

Although many studies indicate that genistein supplementation can improve CVD-related risk factors, several report conflicting results [17]. These inconsistencies highlight the need for a more comprehensive assessment of the effects of genistein on various CVD risk factors [18, 19]. Moreover, existing meta-analyses have not comprehensively addressed all CVD risk factors. The existing analysis [20] only addresses lipid-related risk factors without considering the impact of blood glucose, blood pressure, and inflammatory factors on CVDs. Therefore, further systematic evaluations and meta-analyses are necessary to assess the impact of genistein supplementation on CVD risk factors. This meta-analysis aims to provide an updated assessment of the impact of genistein supplementation on CVD risk factors. The study investigates the effects of genistein consumption as a supplement on body mass index (BMI), lipid profile, blood pressure (systolic pressure (SBP) and diastolic pressure (DBP)), fasting glucose, insulin-related indicators (fasting insulin and homeostatic model assessment of insulin resistance (HOMA-IR)), homocysteine (HCY), and C-reactive protein (CRP) in the overall population. This study aims to demonstrate the benefits of genistein supplementation for CVDs through a meta-analysis of various cardiovascular risk factors, providing a basis for further clinical dosage exploration.

## 2. Methods

This systematic review and meta-analysis of randomized controlled trials (RCTs) was conducted in compliance with the Preferred Reporting Items for Systematic Reviews and Meta-Analyses (PRISMA 2020) statement [21].

### 2.1. Literature Search Strategy

An electronic literature search was performed in the PubMed, Embase, ISI Web of Science, and Cochrane Library databases from inception until June 2024, restricted to publications in English. Two authors independently searched the electronic databases. We used medical subject headings (MeSH) and the following keywords: genistein, CVD, and RCT, along with their related MeSH terms. Additional keywords included BMI, total cholesterol (TC), triglyceride (TG), high-density lipoprotein-cholesterol (HDL-C), low-density lipoprotein-cholesterol (LDL-C), lipoprotein(a) (Lp(a)), blood glucose, blood insulin, homeostasis model assessment of insulin resistance (HOMA-IR), HCY, and CRP. Keywords related to intervention studies included controlled trial, placebo, randomized controlled trial, and double-blind. The search was limited to human studies. Keywords were searched in article titles, abstracts, and hedge words. The specific search protocol can be found in Supporting Table 1. The search results were cross-checked, and any discrepancies were resolved through discussion.

### 2.2. Study Selection

Initially, duplicate publications were excluded, followed by a comprehensive assessment of each study based on the title and abstract to identify studies that potentially fulfilled the eligibility criteria. The authors then conducted a thorough examination of the full text of each paper that passed the initial assessment. All clinical trials assessing the impact of genistein supplementation on the blood lipid profile and other related indicators were incorporated into the meta-analysis. We excluded studies that combined genistein with other supplements to ensure that the effects observed in this meta-analysis were specifically attributable to genistein alone. The primary goal was to isolate the independent impact of genistein supplementation on cardiovascular risk factors, as combining it with other nutraceuticals could introduce confounding variables, complicating the interpretation of the results. To fully isolate the effects of genistein, it is essential to ensure that the included studies treat genistein as a separate intervention factor rather than in combination with other supplements. In addition, we excluded populations such as pregnant or lactating women from our analysis due to the potential risks associated with genistein supplementation during these periods. While genistein may have beneficial effects on cardiovascular health in nonpregnant adults, its impact during pregnancy and lactation remains unclear and may differ due to the physiological changes in these populations [22, 23]. As such, we focused on nonpregnant adults to ensure the safety and reliability of the findings. This exclusion may limit the generalizability of the results to these specific groups, but future studies should address these populations separately to better evaluate the potential effects of genistein in these contexts.

Studies were included if they met the following criteria: (1) conducted in adult individuals, (2) RCTs, (3) genistein prescribed as a single supplement or enrichment, and (4) investigated the effects of genistein on blood lipid profiles and other related indicators. Studies were excluded if they (1) used a combination of genistein with other compounds exclusively in the intervention group, (2) were uncontrolled studies, (3) included pregnant or lactating women, or (4) were animal studies, conference papers, reviews, letters, editorial articles, or case reports.

### 2.3. Data Extraction

Data were gathered from eligible articles using a predesigned, standardized electronic data collection form. The collected information included the last name of the first author, publication year, country of publication, and demographic details of participants such as sex and age, study design (including crossover and parallel study), sample size in both control and intervention groups, type and dosage of intervention in each group, trial duration, and the outcomes investigated, which included BMI, TC, TG, HDL-C, LDL-C, Lp(a), SBP, DBP, fasting glucose, fasting insulin, HCY, and CRP.

### 2.4. Quality Assessment

Data were entered into the Cochrane Review Manager software (RevMan 5.4.1). The quality assessment of the included studies was conducted independently by two authors using the Cochrane Collaboration Scale. This scale comprises various criteria to evaluate the quality of the studies, including (1) adequacy of sequence generation (selection bias); (2) allocation concealment (selection bias); (3) blinding of participants and personnel (performance bias); (4) blinding of outcome assessment (detection bias); (5) incomplete outcome data (attrition bias); (6) selective reporting (reporting bias); and (7) other sources of bias. Following the guidelines outlined in the Cochrane Handbook [24], the studies were assessed and categorized within each domain based on their risk of bias as either low (L), high (H), or unclear (U).

### 2.5. Statistical Analysis

With the exception of SBP, DBP, fasting insulin, and homocysteine, all other blood circulation indicators are reported in milligrams per deciliter (mg/dL). SBP and DBP are denoted in millimeters of mercury (mmHg), fasting insulin is measured in microunits per milliliter (μIU/mL), and homocysteine is expressed in micromoles per liter (μmol/L). HOMA-IR, on the other hand, is a unitless measurement. In both groups, the mean change and standard deviation (SD) at baseline and postintervention were utilized. We computed the net changes by subtracting the control group's changes (final values minus baseline values) from the intervention group's changes in mean values. The effect sizes are presented as weighted mean differences (WMDs) along with corresponding 95% confidence intervals (CIs). The random-effects model (DerSimonian–Laird methodology) was applied to calculate the overall effect sizes to minimize the influence of heterogeneity. To evaluate statistical heterogeneity, we employed Cochran's Q heterogeneity test and calculated the I^2^ statistic. Furthermore, we utilized a funnel plot to identify potential publication bias. We employed Begg's rank test, Egger's test, and funnel plots to assess the presence of potential publication bias [25].

Additionally, we conducted a sensitivity analysis to address the potential impact of using a random-effects model on the width of CIs. As random-effects models tend to yield broader intervals compared to fixed-effect models, the sensitivity analysis aimed to examine the robustness of the treatment impact estimation. By exploring different modeling approaches and comparing the results, we aimed to ensure a comprehensive assessment of the treatment effect while considering the potential variability across studies and providing a more conservative estimation of the treatment impact. R software (version 4.1.0, https://www.R-project.org) with the meta package (version 5.1.1) was used in the data analysis.

## 3. Results

### 3.1. Literature Search

**Figure 1:**
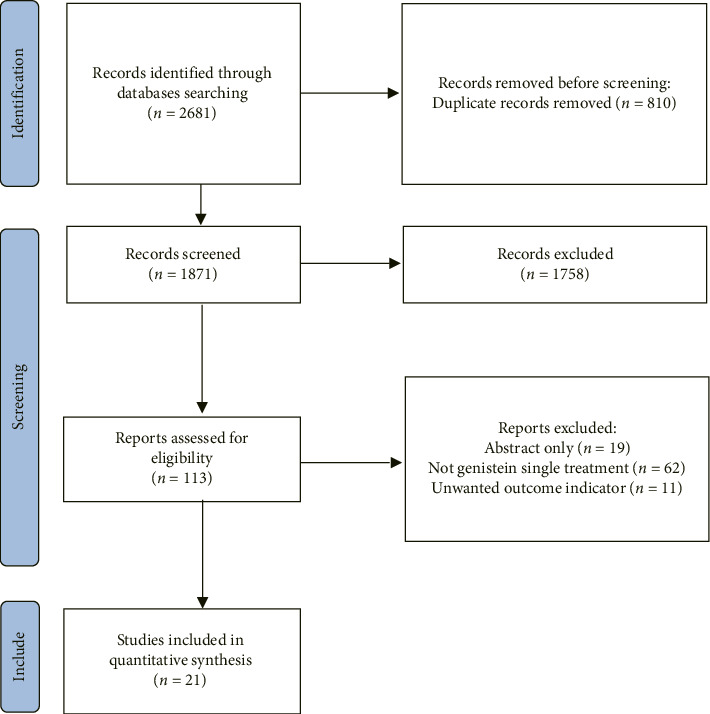
The PRISMA flowchart visually outlines the systematic approach used in this study for conducting literature searches and selecting relevant studies.

Figure 1 illustrates the process of literature screening. A total of 2681 articles were obtained through electronic and manual searches, with 810 duplicates removed. Of the remaining articles, 1758 were excluded after reviewing their titles and abstracts for irrelevance to the impact of genistein supplementation on CVD risk factors. Among the remaining 113 articles, 92 were excluded for not meeting the predefined inclusion and exclusion criteria. Ultimately, 21 eligible RCTs [9, 17, 25–41] were included in the analysis.

### 3.2. Characteristics of Studies and Risk-of-Bias Assessment

**Figure 2:**
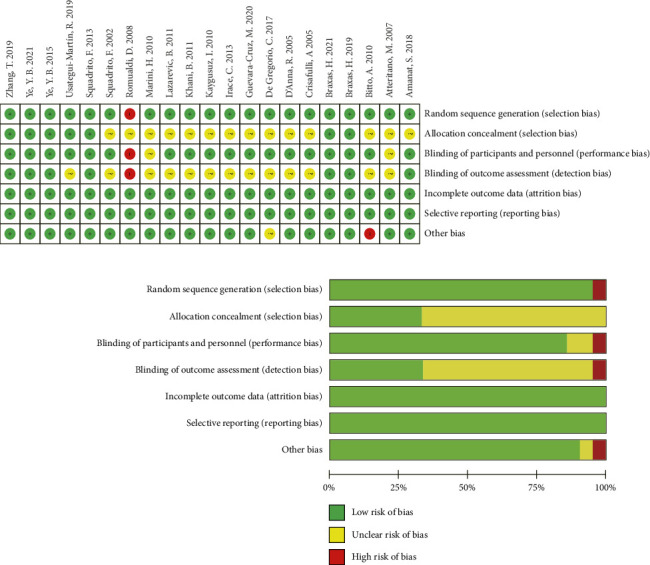
Quality assessment was carried out to ascertain the potential bias risks within the selected randomized controlled trials.

Table 1 presents the pertinent attributes of the 21 trials encompassed in this review. This meta-analysis included a total of 1859 participants, with 941 participants in the genistein supplementation group and 918 participants in the control group. Apart from five studies [26–30] conducted in healthy populations, the remaining studies involved nonhealthy populations, including individuals with metabolic syndrome [9, 10, 32], hyperlipidemia [34], diabetes [39, 40], impaired glucose regulation [18, 38], obesity [31], nonalcoholic fatty liver disease [33], and other pathological states. Across all studies, published between 2002 and 2021, genistein dosages ranged from 30 to 250 mg per day, with study durations varying from 3 weeks to 3 years. For articles utilizing the same baseline data [10, 17, 35, 38, 41], we conducted analyses based on different intervention durations, taking into account the varying number of participants included at different time points. This approach helped in exploring the time dependency of genistein intervention. RCTs were assessed using a “risk-of-bias” assessment tool. The summary and graphical representation of the bias risk are presented in Figure 2 and Supporting Table 2.

### 3.3. Results of Meta-Analysis

#### 3.3.1. Effect on BMI

**Figure 3:**
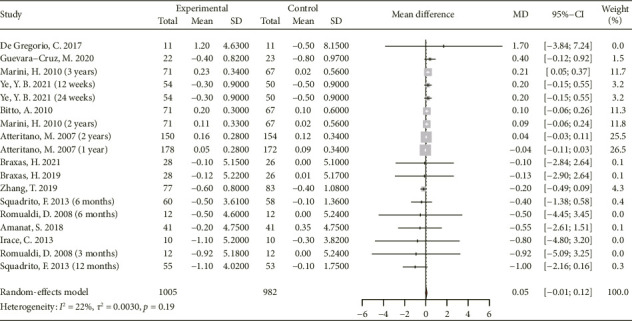
Forest plot of the comparison of the effects of genistein supplementation versus placebo on body mass index.

This study encompassed 13 RCTs, involving 663 participants in the intervention group and 646 in the control group, to assess the relationship between genistein and BMI. The analysis (Figure 3) revealed no statistically significant effect [(MD 95% CI: 0.05 (−0.01, 0.12); *p*=0.19), *I*^2^ = 22%].

#### 3.3.2. Effect on Lipid Profiles

**Figure 4:**
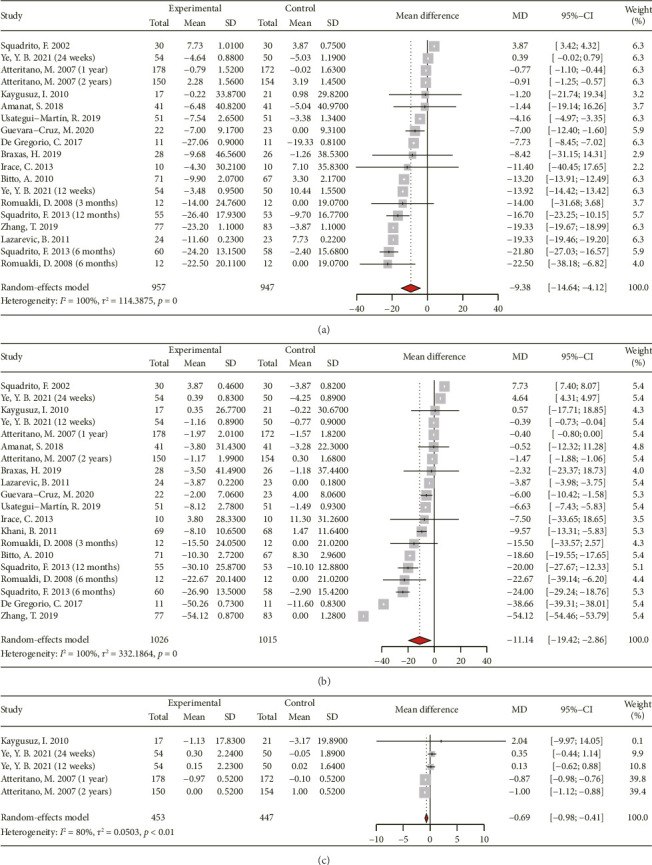
Forest plot of the comparison of the effects of genistein supplementation versus placebo on lipid profile, including total cholesterol (a), low-density lipoprotein-cholesterol (b), and lipoprotein (a) (c).

Figure 4 and Supporting Figure 1 present a forest plot summarizing the effects of genistein supplementation on lipidomic parameters, including TC, TG, HDL-C, LDL-C, and Lp(a). Due to substantial heterogeneity, a random-effects model was employed for all analyses. Fifteen RCTs provided evaluable TC data (686 participants in the experimental group and 678 in the control group). The analysis of 16 studies (755 participants in the experimental group and 746 in the control group) examining the impact of genistein consumption on LDL-C indicated significant reductions. Regarding Lp(a), 249 participants in the experimental group and 243 in the control group from three studies were evaluated. The combination of results from 17 studies (826 participants in the experimental group and 813 in the control group) showed that genistein consumption significantly reduces TC ([MD 95% CI: −9.38 [−14.64, −4.12]; *p* < 0.001], *I*^2^ = 100%), LDL-C ([MD 95% CI: −11.14 [−19.42, −2.86]; *p* < 0.001], *I*^2^ = 100%), and Lp(a) levels ([MD 95% CI: −0.69 [−0.98, −0.41]; *p* < 0.01], *I*^2^ = 80%). However, the combined results of 17 RCTs did not show a statistically significant decrease in HDL-C ([MD 95% CI: 1.68 [−0.83, 4.18]; *p* < 0.001], *I*^2^ = 100%).

#### 3.3.3. Effect on Blood Pressure

**Figure 5:**
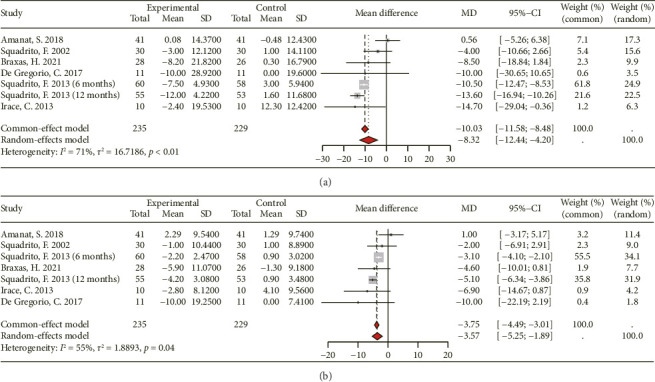
Forest plot of the comparison of the effects of genistein supplementation versus placebo on systolic pressure (a) and diastolic pressure (b).

Blood pressure data from 6 RCTs (180 participants in the experimental group and 176 in the control group) were available for assessment (Figure 5). Given the high heterogeneity (*I*^2^ > 50%) among the RCTs, a random-effects model was employed. The combination of results showed that genistein consumption significantly reduces SBP ([MD 95% CI: −8.32 [−12.44, −4.20]; *p* < 0.01], *I*^2^ = 71%) and DBP ([MD 95% CI: −3.57 [−5.25, −1.89]; *P*=0.04], *I*^2^ = 55%).

#### 3.3.4. Effect on Fasting Glucose

**Figure 6:**
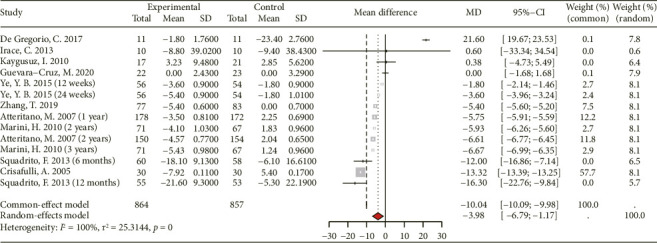
Forest plot of the comparison of the effects of genistein supplementation versus placebo on fasting glucose.

A total of 14 studies involving 532 participants in the intervention group and 529 in the control group were incorporated into the analysis to assess the effect of genistein consumption on fasting glucose levels (Figure 6). The pooled analysis indicates that genistein supplementation significantly reduces fasting blood glucose levels compared to the control group ([MD 95% CI: −3.98 [−6.79, −1.17]; *p* < 0.001], *I*^2^ = 100%).

#### 3.3.5. Effect on Fasting Insulin and HOMA-IR

**Figure 7:**
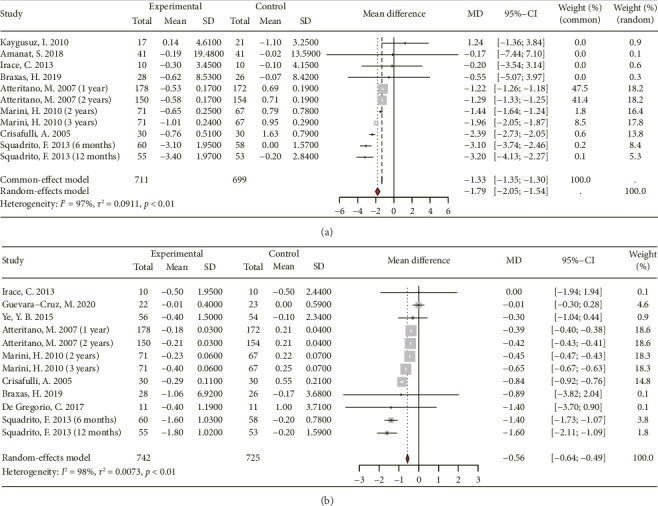
Forest plot of the comparison of the effects of genistein supplementation versus placebo on fasting insulin (a) and homeostasis model assessment of insulin resistance (b).

Data from 11 RCTs with 460 subjects (435 in the experimental group and 425 in the control group) and nine RCTs with 917 subjects (466 in the experimental group and 451 in the control group) were included in the meta-analysis examining the effects of genistein supplementation on fasting insulin and HOMA-IR, respectively (Figure 7). The findings show that genistein consumption significantly reduces fasting insulin ([MD 95% CI: −1.79 [−2.05, −1.54]; *p* < 0.01], *I*^2^ = 97%) and HOMA-IR ([MD 95% CI: −0.56 [−0.64, −0.49]; *p* < 0.01], *I*^2^ = 98%).

#### 3.3.6. Effect on Homocysteine

**Figure 8:**
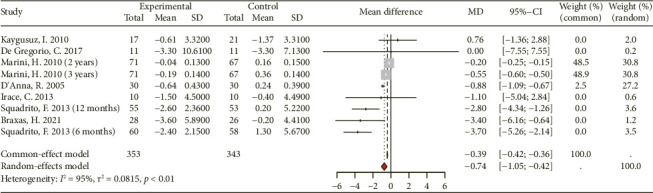
Forest plot of the comparison of the effects of genistein supplementation versus placebo on homocysteine.

Within the scope of this study, the analysis incorporated homocysteine data extracted from 7 RCTs involving 227 participants in the experimental group and 223 participants in the control group (Figure 8). Due to the considerable heterogeneity observed among the included RCTs (*I*^2^ > 50%), the random-effects model was employed for statistical analysis. The findings demonstrate that supplementation with genistein, compared to the placebo group, is significantly associated with a reduction in homocysteine levels ([MD 95% CI: −0.74 [−1.05, −0.42]; *p* < 0.01], *I*^2^ = 95%).

#### 3.3.7. Effect on CRP

**Figure 9:**
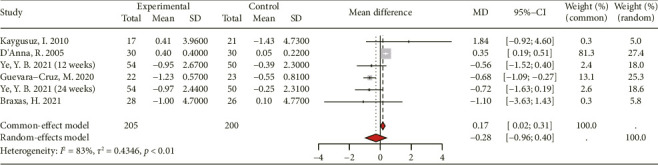
Forest plot of the comparison of the effects of genistein supplementation versus placebo on C-reactive protein.

The analysis included a total of 5 studies, comprising 151 participants in the intervention group and 150 participants in the control group, to evaluate the impact of genistein consumption on CRP levels (Figure 9). Due to substantial heterogeneity observed among the included RCTs (*I*^2^ = 83%), the random-effects model was employed for statistical analysis. The aggregated findings did not yield statistically significant evidence of an effect of genistein consumption on CRP ([MD 95% CI: −0.28 [−0.96, 0.40]; *p* < 0.01]).

#### 3.3.8. Dose–Response Relationships

**Figure 10:**
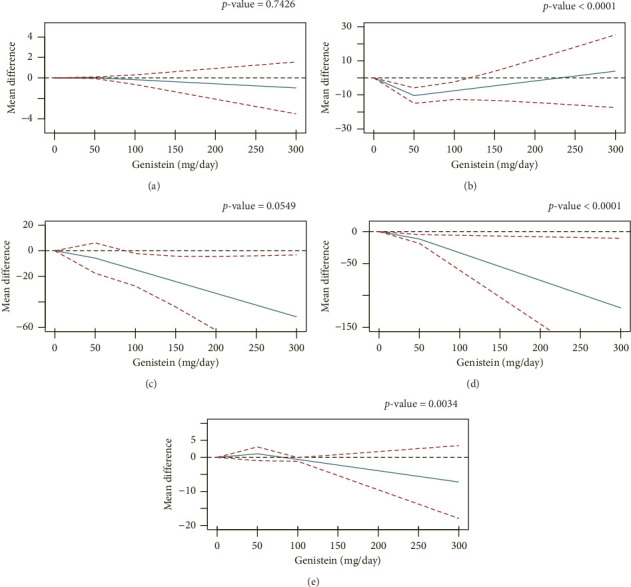
Dose–response relationship curve of body mass index (a), total cholesterol (b), triglyceride (c), low-density lipoprotein cholesterol (d), and high-density lipoprotein cholesterol (e).

Due to the constraints imposed by the prerequisites for establishing dose–response relationships, this study was limited to constructing dose–response curves for specific indicators, including BMI and certain lipidomic parameters (TG, TC, HDL-C, LDL-C) (Figure 10). Apart from BMI (*χ*^2^ = 0.5953, *p* = 0.7426) and TG (*χ*^2^ = 5.8062, *p* = 0.0549), statistically significant dose–response curves were observed between genistein supplementation and the remaining indicators (TC [*χ*^2^ = 20.1235, *p* < 0.001], HDL-C [*χ*^2^ = 11.3532, *p* < 0.001], LDL-C [*χ*^2^ = 4340.22, *p* < 0.001]). The dose–response curve of genistein supplementation with TC exhibited a J-shaped pattern, with the lowest point observed at approximately 50 mg/day. This suggests that the optimal effective dose of genistein for reducing TC is around 50 mg/day (Figure 10(b)). The dose–response curve analysis for HDL-C and genistein supplementation exhibited a similar pattern to that of TC, but with an opposite trend in terms of changes (Figure 10(e)). The dose–response curve analysis revealed a negative correlation between genistein supplementation and LDL-C levels, suggesting that higher doses of genistein were associated with a reduction in LDL-C levels (Figure 10(d)).

#### 3.3.9. Publication Bias and Sensitivity Analysis

Visual inspection of the funnel plot (Supporting Figures 2–4) was employed to detect potential publication bias. Publication bias in the meta-analysis of genistein supplementation's impact on BMI, TC, TG, HDL-C, LDL-C, fasting glucose, fasting insulin, and HOMA-IR was examined using Egger's regression asymmetry tests and Begg's tests. Apart from fasting glucose and HOMA-IR, the remaining results did not indicate the presence of publication bias, which may be a source of heterogeneity in the effects of genistein supplementation on fasting glucose and HOMA-IR. Due to the limited number of included studies (*k* < 10), it was not feasible to perform Begg's test or Egger's test to assess the impact of genistein supplementation on SBP, DBP, Lp(a), HCY, and CRP. In addition, the sensitivity analysis revealed that the exclusion of each individual study did not result in significant changes to the overall effect sizes for all parameters, except for HDL-C, Lp(a), and CRP (Supporting Figures 5–12).

## 4. Discussion

In this meta-analysis, we conducted a comprehensive assessment of the effects of genistein supplementation on CVD-related risk factors. Our study demonstrates that genistein supplementation leads to significant reductions in TC, LDL-C, Lp(a), blood pressure (SBP and DBP), fasting glucose, insulin, HOMA-IR, and HCY levels. It is well known that reductions in indicators such as TC, LDL-C, and SBP can significantly reduce the risk of CVD [42]. Although not statistically significant, genistein supplementation also resulted in decreases in TG and CRP levels, as well as an increase in HDL-C. This may be due to the small number of RCT studies included for these indicators, differences in study designs (such as variations in genistein dosage, baseline health status, and intervention duration), or methodological factors (e.g., small sample sizes, insufficient dosage, or short follow-up periods), which led to the loss of significance in the results.

Our findings are consistent with previous research [20] on the effects of genistein intake on cardiovascular risk factors. However, our findings regarding blood pressure contradict a previous meta-analysis [43], which reported no significant effects on SBP and DBP. These discrepancies may be attributed to variations in the number of included studies, as well as differences in the duration and dosage of interventions. Subgroup analysis of the study [43] also revealed that among participants with metabolic syndrome, supplementation with genistein for 6 months or longer significantly decreased SBP and DBP levels, mirroring our study results. This indicates that the duration of genistein intervention is crucial in influencing blood pressure.

We conducted subgroup analyses to examine the impact of treatment duration, geographic region, and participant health status on the effects of genistein supplementation. However, the analysis of key factors such as age, gender, and dosage was limited. The age and gender distribution in the included studies was relatively homogeneous, with most participants being women around 50 years of age, making subgroup analyses based on these variables less feasible. Furthermore, the majority of the studies used a dosage of 54 mg/day, with a few studies reporting higher or lower dosages. Given this limited variation in dosage, a more in-depth subgroup analysis for dosage was not conducted. Future research with a wider range of participant ages, gender distributions, and dosage variations would be valuable in exploring these additional factors.

Significant heterogeneity was observed in the effects of genistein on indicators other than BMI. Regarding the statistical analysis methods, we used a random-effects model due to the observed high heterogeneity in the data. Given the variation in factors such as genistein dosage, study design, and participant health status, the random-effects model offers a more robust framework for interpreting the results. Subgroup analysis did not identify any significant differences, suggesting that the heterogeneity was not influenced by factors such as treatment duration, geographic region, or participant health status. However, it should be noted that sensitivity analysis, which involved the removal of certain studies, yielded opposite results for HDL-C [36], Lp(a) [17], and CRP [27]. Consequently, the heterogeneity for these indicators may originate from the influence of the excluded literature.

The dose–response relationship analysis showed that increasing genistein intake resulted in a downward trend in the levels of BMI, TG, and LDL-C. The dose–response relationship for TC and HDL-C exhibited a J-shaped curve, with a turning point at approximately 50 mg/day. Below this dosage, increasing genistein supplementation led to a decrease in TC levels and an increase in HDL-C levels. However, higher dosages (> 50 mg/day) were associated with higher TC levels and lower HDL-C levels. In summary, genistein supplementation has a protective effect on blood lipid levels, with the optimal protective dosage for TC and HDL-C levels appearing to be around 50 mg/day, aligning with typical dosages used in most RCTs. To improve clinical application, we recommend that genistein supplementation be considered for populations such as postmenopausal women, individuals with hypertension, and those with metabolic syndrome, as these groups may derive the most benefit from its effects. Based on the findings from our meta-analysis as well as previous reports [9, 10, 17], an approximate dosage regimen of 50 mg/day is suggested for optimal efficacy, although further clinical trials are needed to fine-tune dosage recommendations for specific populations.

Previous meta-analyses [44, 45] indicated that among the soy isoflavones, only genistein has a significant impact on improving glucose metabolism. Our study supports this, demonstrating that genistein lowers blood glucose and insulin levels while improving insulin sensitivity. However, previous studies encountered issues such as duplicate use of the same RCT and inclusion of only postmenopausal women [44, 45]. In contrast, our study includes a sample population covering all age groups, providing a more comprehensive evaluation of genistein's effects on blood glucose, insulin levels, and insulin sensitivity.

Since genistein is predominantly obtained through dietary sources [46], the dietary patterns of participants (e.g., Mediterranean, plant-based, and Nordic) can significantly influence the effects of genistein supplementation on CVD risk factors [47]. The lack of precise information on patients' dietary patterns in the included RCTs may contribute to potential bias in interpreting the results. Moreover, recent studies have indicated that dietary habits (such as soy-derived foods) and the effects of genistein supplementation on CVD markers may be associated with the composition of the gut microbiome [48, 49]. In future research, more attention should be focused on the potential interactions between gut microbiome profiles and genistein's cardiometabolic effects. Furthermore, studies have shown that genistein intervention combined with exercise can significantly improve diabetes-induced cardiac complications in ovariectomized rats [50]. In humans, regular physical activity may similarly influence the effects of genistein supplementation on cardiovascular health [51, 52]. Therefore, variations in exercise habits among participants may also lead to biases in the results. In light of the influence of the aforementioned factors and insights from previous research [47, 51], we may consider implementing a combined management approach in clinical settings that incorporates a healthy diet, nutraceuticals (such as genistein), and exercise interventions. This integrated strategy aims to mitigate a range of risk factors associated with CVDs through comprehensive lifestyle modifications [53]. By addressing multiple pathways simultaneously, such combinations could potentially enhance therapeutic outcomes and provide a more robust framework for the prevention and management of noncommunicable diseases.

Regarding the applicability of the results, since the geographical locations of the included RCTs are primarily limited to European and Asian countries, the findings may not be fully generalizable to other countries and regions. This indicates limitations in terms of nationality and ethnicity, affecting representativeness. It also suggests that future studies in different countries and regions be conducted to enrich the findings and enhance their general applicability. In addition, research has shown that the PPAR gene is associated with TC, LDL-C, and some inflammatory markers [54], and the activation of PPAR-α and PPAR-γ facilitates cholesterol clearance [55]. Therefore, the observed reductions in cholesterol indicators and validated biomarker levels may be related to these mechanisms, which require further investigation.

Overall, our study provides a more comprehensive and accurate evaluation compared to previous meta-analyses. We included RCTs that used pure genistein as the sole intervention measure, incorporated a diverse sample population, and covered a wide range of cardiovascular risk factors. However, some limitations should be acknowledged in this study. Most of the analyzed indicators showed substantial heterogeneity, potentially due to differences in study design, participant characteristics, intervention measures, and outcome assessments. Additionally, genistein, being an estrogen-like substance, was predominantly studied in postmenopausal women in the included RCTs. This uneven distribution of gender and age introduces a potential risk of bias in the findings.

## 5. Conclusion

In summary, our meta-analysis demonstrates that genistein can significantly improve TC, LDL-C, Lp(a), blood pressure (SBP and DBP), fasting blood glucose and insulin, HOMA-IR, and HCY levels. However, no significant improvement was observed in BMI, TG, HDL-C, and CRP. Additionally, the decrease in TG and LDL-C levels is positively correlated with the dosage of genistein supplementation, indicating that higher dosages yield better results. Further research should focus on elucidating the molecular mechanisms underlying the beneficial metabolic effects of genistein supplementation, particularly in populations with hypertension, diabetes, or metabolic syndrome. Understanding these mechanisms will provide valuable insights into the metabolic benefits of genistein supplementation and help optimize its use for cardiovascular health.

## Figures and Tables

**Table 1:** Characteristics of the included studies.

Trial	Country	Design	Dosage (mg/d)	Duration	Age (intervention, control)	Gender (M/F)	Sample size (intervention/control)
Amanat 2018	Iran	Parallel	250	8 weeks	44.22 ± 11.80 42.94 ± 9.55	30/11 31/10	82 (41/41)
Atteritano 2007	Italy	Parallel	54	1 year 2 years	54.70 ± 0.25 54.20 ± 0.19	0/350	350 (178/172)
Bitto 2010	Italy	Parallel	54	3 years	49–67	0/138	138 (71/67)
Braxas 2021	Iran	Parallel	54	12 weeks	57.92 ± 5.72 57.38 ± 6.54	0/54	54 (28/26)
Braxas 2019	Iran	Parallel	54	12 weeks	57.92 ± 5.72 57.38 ± 6.54	0/54	54 (28/26)
Crisafulli 2005	Italy	Parallel	54	6 months	54.00 ± 1.28 57.00 ± 1.09	0/60	60 (30/30)
D'Anna 2005	Italy	Parallel	54	6 months	50–60	0/60	60 (30/30)
De Gregorio 2017	Italy	Parallel	54	12 months	55.00	0/22	22 (11/11)
Guevara-Cruz 2020	Mexico	Parallel	50	2 months	42.60 ± 1.90 43.00 ± 2.28	NR	45 (22/23)
Irace 2013	Italy	Parallel	54	6 months	60.10 ± 5.90 57.50 ± 8.60	0/20	20 (10/10)
Kaygusuz 2010	Turkey	Parallel	50	6 months	NR	0/38	38 (21/17)
Khani 2011	Iran	Parallel	36	3 months	27.11 ± 5.76 27.45 ± 5.77	0/137	137 (69/68)
Lazarevic 2011	Norway	Parallel	30	3–6 weeks	60.70 ± 1.07 58.40 ± 1.73	47/0	47 (24/23)
Marini 2010	Italy	Parallel	54	2 years 3 years	53.80 ± 0.34 53.50 ± 0.24	0/138	138 (71/67)
Romualdi 2008	Italy	Parallel	36	3 months 6 months	23.33 ± 4.80	0/24	24 (12/12)
Squadrito 2002	Italy	Parallel	54	6 months	54.00 ± 7.00 58.00 ± 6.00	0/60	60 (30/30)
Squadrito 2013	Italy	Parallel	54	6 months 12 months	55.60 ± 4.60 55.40 ± 4.80	0/108	108 (55/53)
Usategui-Martín 2019	America	Parallel	90	12 weeks	55.42 ± 3.81	0/102	102 (51/51)
Ye 2014	China	Parallel	50	12 weeks 24 weeks	57.00 ± 9.68 56.30 ± 11.10	0/110	110 (56/54)
Ye 2021	China	Parallel	50	12 weeks	57.00 ± 9.68 56.30 ± 11.10	0/104	104 (54/50)
Zhang 2019	China	Parallel	60	6 months	57.20 ± 5.20 56.70 ± 4.90	0/160	160 (77/83)

Abbreviations: mg/d, milligram per day; NR, not reported.

## Data Availability

Data sharing is not applicable to this article as no datasets were generated or analyzed during the current study.

## Supporting Information

The Supporting Information contains Supporting Figures 1–12 and Supporting Table 1–2, which have been clearly described in manuscript.

Supporting Figure 1: Forest plot of the comparison of the effects of genistein supplementation versus placebo on lipid profile, including TG (A) and HDL-C (B).

Supporting Figure 2: The funnel plot analysis indicated the presence of publication bias in the studies examining the effects of genistein supplementation on some cardiovascular risk factors including body mass index (A), triglyceride (B), total cholesterol (C), low-density lipoprotein-cholesterol (D), high-density lipoprotein-cholesterol (E), and lipoprotein(a) (F).

Supporting Figure 3: The funnel plot analysis indicated the presence of publication bias in the studies examining the effects of genistein supplementation on some cardiovascular risk factors including SBP (A) and DBP (B).

Supporting Figure 4: The funnel plot analysis indicated the presence of publication bias in the studies examining the effects of genistein supplementation on some cardiovascular risk factors including fasting glucose (A), fasting insulin (B), homeostasis model assessment of insulin resistance (C), homocysteine (D), and C-reactive protein (E).

Supporting Figure 5: Sensitivity analysis of body mass index.

Supporting Figure 6: Sensitivity analysis of total cholesterol (A), low-density lipoprotein-cholesterol (B), and lipoprotein(a) (C).

Supporting Figure 7: Sensitivity analysis of triglyceride (A) and high-density lipoprotein-cholesterol (B).

Supporting Figure 8: Sensitivity analysis of systolic pressure (A) and diastolic pressure (B).

Supporting Figure 9: Sensitivity analysis of fasting glucose.

Supporting Figure 10: Sensitivity analysis of fasting insulin (A) and homeostasis model assessment of insulin resistance (B).

Supporting Figure 11: Sensitivity analysis of homocysteine.

Supporting Figure 12: Sensitivity analysis of C-reactive protein.

Supporting Table 1: Database Search Operations.

Supporting Table 2: Specific examples of the studies rated as risk of bias.
