# Supplementary material for: Improvement of Cardiovascular Risk Factors by Genistein Supplementation: A Systematic Review and Meta-Analysis in Diverse Population-Based RCTs
Source: J Nutr Metab. 2025 Mar 18;2025:1827252. doi: 10.1155/jnme/1827252 (PMC11936529; doi:10.1155/jnme/1827252)
Supplement: Supporting Information — Additional supporting information can be found online in the Supporting Information section. [file 1827252.f1.zip › supplemental table.docx]

**Supplemental Table 1. Database Search Operations**

| Database | Search Strategy |
| --- | --- |
| *PubMed, ISI Web of Science, and Cochrane Library* | (genistein) AND ((lipid) OR (cholesterol) OR (TC) OR (Triglyceride) OR (TG) OR (high-density lipoprotein) OR (HDL) OR (low-density lipoprotein) OR (LDL) OR (body mass index) OR (BMI) OR (blood pressure) OR (systolic blood pressure) OR (SBP) OR (diastolic blood pressure) OR (DBP) OR (body weight) OR (Apolipoprotein) OR (Apo) OR (lipoprotein) OR (Apolipoprotein B) OR (Apo B) OR (Apolipoprotein A1) OR (Apo A1) OR (LP(a))OR (glucose) OR (GLU) OR (insulin) OR (RI) OR (HOMA-IR) OR (IRI) OR (Insulin resistance index) OR (CRP) OR (C reaction protein) OR (C-reaction protein) OR (Homocysteine) OR (HCY)) AND ((Intervention Studies) OR (intervention) OR (controlled trial) OR (randomized) OR (randomly) OR (placebo) OR (assignment) OR (randomized controlled trial) OR (randomized clinical trial) OR (RCT) OR (blinded) OR (double blind) OR (double blinded) OR (trial) OR (controlled clinical trial) OR (pragmatic clinical trial) OR (crossover procedure) OR (cross-over trial) OR (double-blind method) OR (equivalence trial) OR (double blind procedure)) |
| *Embase* | ('genistein'/exp OR genistein) AND ('lipid':ab OR 'cholesterol':ab OR 'tc':ab OR 'triglyceride':ab OR ‘tg':ab OR 'high-density lipoprotein':ab OR 'hdl':ab OR 'low-density lipoprotein':ab OR 'ldl':ab OR 'body mass index':ab OR 'bmi':ab OR 'blood pressure':ab OR 'systolic blood pressure':ab OR 'sbp':ab OR 'diastolic blood pressure':ab OR 'dbp':ab OR 'body weight':ab OR 'apolipoprotein':ab OR 'apo':ab OR 'lipoprotein':ab OR 'apolipoprotein b':ab OR 'apo b':ab OR 'apolipoprotein a1':ab OR 'apo a1':ab OR 'lp(a)':ab OR 'glucose':ab OR 'GLU':ab OR 'insulin':ab OR 'RI':ab OR ‘HOMA-IR':ab OR 'IRI':ab OR 'Insulin resistance index':ab OR 'CRP':ab OR 'C reaction protein':ab OR 'C-reaction protein':ab OR 'Homocysteine':ab OR 'HCY':ab) AND ('intervention studies':ab OR 'intervention':ab OR 'controlled trial':ab OR 'randomized':ab OR 'randomly':ab OR 'placebo':ab OR 'assignment':ab OR 'randomized controlled trial':ab OR 'randomized clinical trial':ab OR 'rct':ab OR 'blinded':ab OR 'double blind':ab OR 'double blinded':ab OR 'trial':ab OR 'controlled clinical trial':ab OR 'pragmatic clinical trial':ab OR 'crossover procedure':ab OR 'cross-over trial':ab OR 'double-blind method':ab OR 'equivalence trial':ab OR 'double blind procedure':ab) |

**Supplemental Table 2. Specific examples of the studies rated as risk of bias.**

| Examples | Rating Criteria | Example |
| --- | --- | --- |
| Random sequence generation | random allocation or not | High: Romualdi, D. 2008  Low: Amnat, S. 2018 |
| Allocation concealment | conceal allocation information or not | Unknow: Amnat, S. 2018  Low: Braxas, H. 2019 |
| Blinding of participants and personnel | participants and personnel aware of the allocation scheme or not | High: Romualdi, D. 2008  Unknow: Atteritano, M. 2007  Low: Amnat, S. 2018 |
| Blinding of outcome assessment | investigator aware of the results of each group or not | High: Romualdi, D. 2008  Unknow: Atteritano, M. 2007  Low: Amnat, S. 2018 |
| Incomplete outcome data | the result data complete or not | Low: Amnat, S. 2018 |
| Selective reporting | certain data omitted or not | Low: Amnat, S. 2018 |
| Other bias | the baseline balanced, etc. | High: Bitto, A. 2010  Unknow: De Gregorio, C. 2017  Low: Amnat, S. 2018 |
